# Alterations in the expression of DEAD-box and other RNA binding proteins during HIV-1 replication

**DOI:** 10.1186/1742-4690-1-42

**Published:** 2004-12-08

**Authors:** Vyjayanthi Krishnan, Steven L Zeichner

**Affiliations:** 1 HIV and AIDS Malignancy Branch, National Cancer Institute, National Institutes of Health, Building 10, Room 10S255 MSC1868, Bethesda, MD 20892 USA

## Abstract

Recent results showed that certain DEAD box protein RNA helicases, DDX3 and DDX1, play an important role in the HIV infection cycle by facilitating the export of long, singly spliced or unspliced HIV RNAs from the nucleus via the CRM1-Rev pathway. Close examination of an extensive microarray expression profiling dataset obtained from cells latently infected with HIV induced to undergo lytic viral replication indicated that additional DEAD box proteins, beyond DDX3 and DDX1, exhibit differential expression during lytic HIV replication, and in latently infected cells prior to induction into active replication. This finding provides additional evidence that the involvement of DEAD box proteins and other RNA-binding proteins may play roles in active HIV replication and in the control of viral latency. Agents targeting these functions may offer new approaches to antiretroviral therapy and the therapeutic manipulation of HIV latency.

## Findings

The DEAD box proteins, a family of RNA helicases containing the conserved amino acid motif Asp-Glu-Ala-Asp (D-E-A-D in the single letter amino acid code), play an essential role in many aspects of cellular RNA metabolism (reviewed in [1,2]), including RNA transport, transcription, spliceosome function, ribosome assembly, the initiation of translation, and RNA degradation. The HIV Rev protein regulates a key aspect of the HIV replication cycle by mediating the switch between the early pattern of HIV gene expression, in which short, multiply spliced messages encoding the viral regulatory genes Tat, Rev, and Nef are exported from the nucleus, and the late pattern of viral gene expression in which larger singly spliced and unspliced messages that encode the viral structural proteins and that constitute the RNA genomes of progeny virions are exported from the nucleus [3-5]. Recent work, reviewed in reference [6], from the Jeang [7] and Pomerantz [8] laboratories implicate the DDX3 and DDX1 DEAD box proteins as additional critical co-factors for the Rev-mediated export of the long HIV singly spliced and unspliced mRNAs.

In a directed analysis of a large data set, which describes global changes in cellular gene patterns before and after a latently infected cell line was induced into active viral replication [9], we found that genes encoding many DEAD box proteins, and other RNA and DNA binding and modification proteins, in addition to DDX3 and DDX1, showed differential regulation, suggesting that HIV replication may be associated with generalized changes in the expression of many DEAD box proteins and other RNA binding proteins.

Yedavalli et al. [7] identified DDX3 in a differential display-based screen for cellular messages upregulated in the presence of HIV Tat. They found that DDX3 binds the nuclear export protein CRM1, which is essential for nuclear export mediated by the HIV Rev protein [10,11], shuttles between the cytoplasm and nucleus, facilitates the nuclear export of RRE-containing RNA in the presence of Rev, and advances HIV replication. Fang et al. [8] identified DDX1 in a two hybrid screen as a Rev- and RRE-binding protein that enhanced HIV replication, improved the expression of RRE-containing RNA, and modified the subcellular distribution of Rev from a predominantly nuclear to a predominantly cytoplasmic distribution.

Our work on large scale expression profiling in HIV actively and latently infected cells has been guided by the hypothesis that there is one set of cellular conditions which is ideal for normal cellular growth and homeostasis, that there is another set of conditions which may be better suited to supporting viral replication, and that HIV has evolved ways of altering the host cell so as to better support viral replication. An interesting corollary of the hypothesis is that targeting the products of the differentially expressed genes may inhibit viral replication by making the host cell environment less hospitable to viral replication (reviewed in [12,13]). Several laboratories have conducted large scale expression profiling studies investigating changes in cellular gene expression during HIV replication [14-16], and have, in some cases, identified new potential targets for antiviral therapy. The observations that altering the intracellular environment by, for example, targeting kinases involved in signal transduction and cell cycle regulation inhibits HIV replication lends additional support for this hypothesis [17,18]. Investigators studying other viruses, for example Kaposi's sarcoma-associated herpesvirus, have also noted changes in host cell gene expression patterns that accompany infection and transformation [19,20], and have identified targets for potential therapeutic intervention based on those studies. In examining our data, we therefore try to note interesting and potentially targetable host cell genes that show alterations in expression during HIV replication and latency. In our earlier study, we identified cellular genes encoding proteins that constituted new targets for agents aimed at activating latently infected cells into active viral replication [9]. In our initial examination of our dataset, among the several classes of genes showing discrete, temporally-dependent changes in expression during lytic replication, we noticed that several DDX genes and genes encoding other factors involved in RNA metabolism were differentially expressed. However, prior to the work by Yedavalli et al. and Fang et al., we had no clear sense of how the differential expression of those genes might contribute to facilitating HIV replication. Those recent studies prompted us to undertake a more detailed analysis of our data.

In our study, we compared RNA samples obtained from HIV latently infected cell lines prior to induction of active replication by the integrated HIV-1 provirus and following such induction with phorbol myristyl acetate (PMA). We profiled ACH-2 cells treated with PMA side by side with similarly treated HIV-1 naïve parental A3.01 cells to assess differential cellular gene expression patterns associated with HIV lytic replication. The dataset was generated from samples obtained from three independent biological replicate experiments and at least two microarray hybridizations were done for each time point from each biological replicate (for a minimum of 6 microarrays per time point). A detailed description of the cell culture, induction, RNA isolation, and microarray labeling and hybridization methods are contained in reference [9]. Following microarray data acquisition, data were analyzed using commercial (GenePix Pro software, Axon Instruments) and in-house software (microarray database system (mAdb), hosted by Center for Information Technology, NIH). Using BRB -ArrayTools , the data were subjected to statistical analyses using univariate parametric and multivariate permutation analyses, based on the one sample random variance t-statistic, where significance was based on P < 0.001 and the proportion of false discoveries was limited to 0.10 with a 90% confidence level. 1740 genes showed differential gene expression at a minimum of one timepoint during lytic replication. Hierarchical clustering analyses were performed using mAdb clustering tools, as well as Treeview . Since the data was obtained from latently infected cell lines, there may be some concern that infected primary cells may behave in a somewhat different fashion. However, the advantages of using latently infected cell lines are significant: Activation into active replication is reasonably synchronous and includes essentially all the cells, so that the signal from the cells supporting active replication is not diluted by the signal from uninfected cells or cells with virus at different stages of viral replication. Also, the signal comes only from infected cell and not from cells responding to effects from the exposure to very large numbers of defective viral particles that are in high multiplicity of infection inocula.

**Figure 1:**
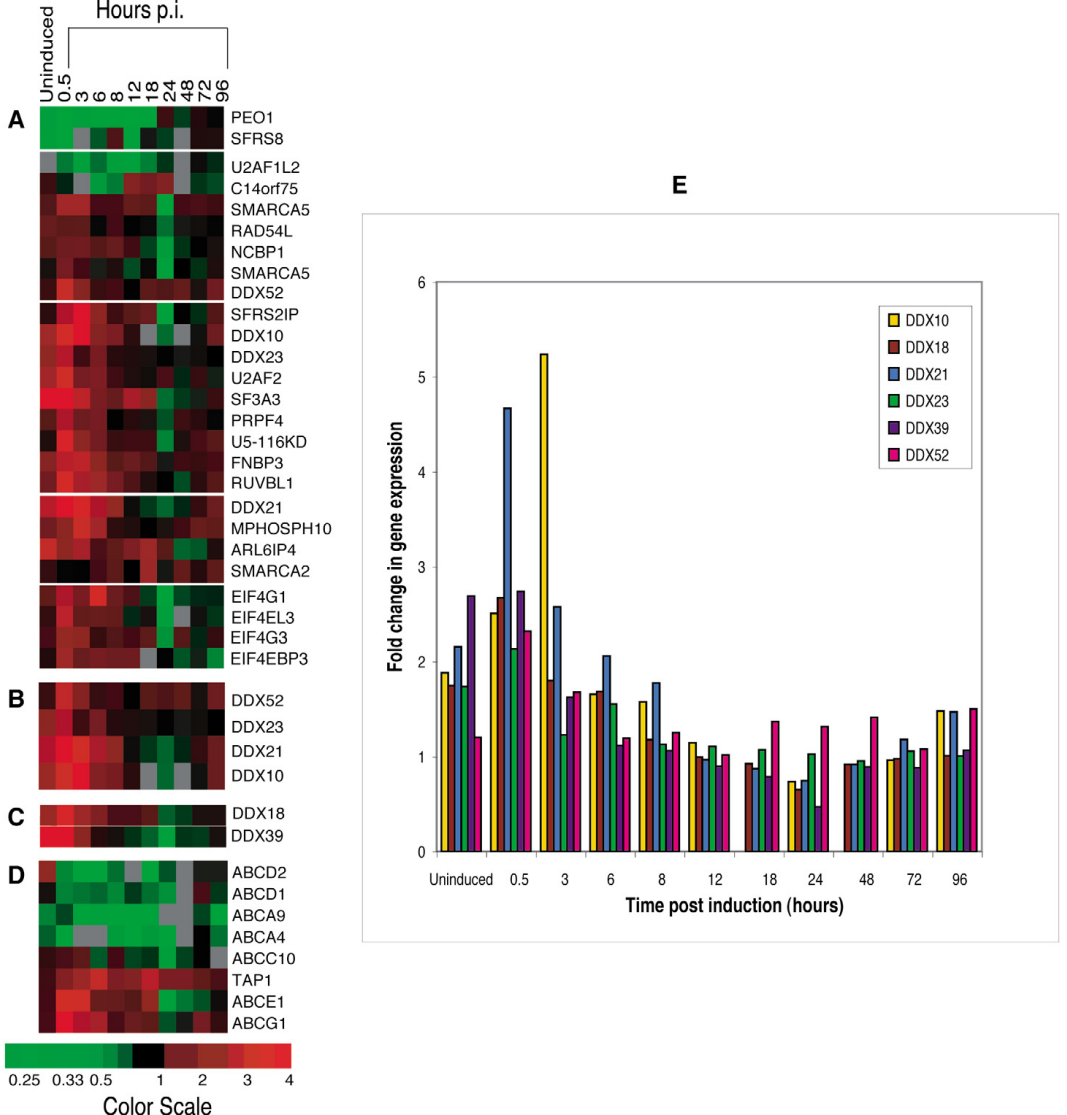
A compilation of the expression profiles of genes with known or putative involvement in RNA binding, transport or splicing before and after latently infected ACH-2 cells were induced into active replication. Panel A shows selected genes involved in RNA metabolism that were differentially expressed during active replication in the infected cells (ACH-2 cells) at 0.5–8 hr post-induction (p.i.) compared to similarly induced, parental uninfected A3.01 cells. Panel B shows gene expression profiles of a subset of DEAD-box proteins, following induction into active replication, of the genes displayed in panel A. Only genes that passed the criteria for statistical significance (P < 0.001) for the 0.5–8 hr p.i. time period (but not for other time periods) are shown. Panel C shows the expression profiles of genes encoding DDX18 and DDX39, which were up regulated during viral latency and during latency and early lytic replication. Panel D shows the expression profile of genes encoding ABC transporter proteins. The bottom of the figure shows a scale indicating the color values corresponding to the expression ratio in HIV infected/HIV uninfected cells for the differential expression of each gene shown in the figure at the different time points. Panel E is a graphical representation of the expression patterns observed in the selected DEAD-box proteins showing fold change in gene expression over corresponding controls.

Figure 1 shows the expression patterns for genes encoding DEAD box proteins, and other genes encoding RNA helicases and RNA binding proteins, as assigned by the gene ontology database [21]. We found that a number of DEAD-box proteins were significantly up regulated (P < 0.001) immediately following induction (0.5 hr post induction p.i). These included *DDX10*, a DEAD box protein with expression in many tissues having tumorigenic activity when fused to the nucleoporin NUP98 [22,23], *DDX21*, a DEAD-box protein originally identified as a nucleolar protein thought to be involved in ribosomal RNA metabolism[24], *DDX23*, a DEAD box protein first identified in U5 SnRNPs with significant homology to the yeast Prp28p splicing factor[25], and *DDX52*, a human DEAD box protein identified through its homologies to a yeast gene [26].

Other genes encoding proteins with RNA splicing/binding and RNA transport activity were also up regulated during this period, including the methylated mRNA cap binding proteins *EIF4G1 *[27] and *NCBP1 *(*CBP80*) [28], which function in translation initiation and may also be involved in mediating nuclear export of RNA. In addition, genes encoding other proteins involved in nucleic acid-protein interactions were also upregulated, such as members of the SWI/SNF family of ATP-dependent chromatin remodeling factors involved in cell cycle control and the regulation of gene expression, *SMARCA2 *and *SMARCA5 *[29,30]. Another class of genes that shows differential expression during the early time period (0.5–8 hr p.i.) are the genes encoding the ATP-dependent ABC transporter proteins, which share sequence homology with members of the helicase family at the ATP binding site [31], indicating that many ATP-dependent processes may be targeted by early viral replication steps, not only as a means to facilitate viral RNA transport but also as a mechanism to shut off or divert cellular functions requiring ATP hydrolysis.

Our findings that several DEAD box protein genes are upregulated during HIV replication lend support to the published finding that two cellular DEAD-box proteins, including one (DDX3) that was identified in a broad-based screen for differentially expressed genes [7], may be important mediators for Rev-mediated RNA export. Our data also show that several other RNA binding proteins are differentially regulated during HIV-1 replication, suggesting that there may be a general involvement of these classes of genes in the HIV replication cycle, that the involvement is not limited only to DDX3 and DDX1. The additional DEAD box family members and other proteins involved in RNA metabolism may be interesting candidates for further mechanistic studies on HIV replication. For example, EIF4G1 has been shown to interact with CBP80 (NCBP1) [32], as well as with EIF4A [33], an RNA helicase with a DEAD-box motif in its sequence. The binding of EIF4G1 to EIF4A is essential for the proper function of EIF4A as an RNA helicase [34]. In our study, genes encoding EIF4G1 and CBP80 were differentially expressed during early lytic replication. Further study of the interactions of EIF4G1, CBP80 and EIF4A1 may thus be important in elucidating Rev function and viral RNA export, as well as the synthesis of viral proteins. While some of the differentially expressed DEAD box proteins, beyond DDX1 and DDX3, may play a part in Rev-dependent viral RNA export from the nucleus, it is also possible that the broad induction of the expression of DEAD box protein-encoding genes and genes encoding other RNA binding factors may indicate that such gene products are involved in other aspects of HIV replication. These aspects of HIV replication could involve activities in which the DEAD box proteins have already been implicated, such as transcription, spliceosome assembly, and translation.

In our recent publication [9], we showed that several host cell genes were differentially expressed in latently infected cell lines, even before induction of the integrated virus into active replication. In an approach analogous to our hypotheses concerning the involvement of cellular genes in active viral replication, we showed that targeting the products of some cellular genes differentially expressed in the latently infected cells could activate viral replication, ejecting the virus from latency. In our examination of the DEAD box proteins, we noted that two genes encoding DEAD box proteins, DDX18, a DEAD-box protein induced by Myc and Max [35] and DDX39, (or URH49), a DEAD-box protein induced by growth stimulation or protein synthesis inhibition thought to be involved in splicing and nuclear export, with homology to the yeast Sub2p protein [36], were differentially expressed during viral latency (DDX39, DDX18) and at early times (DDX18) after induction into active replication. Since some DEAD box proteins are important for viral RNA nuclear export and active viral replication, it may be reasonable to consider that other members of this family could have natural inhibitory activity for HIV replication, such as that seen with mutated DDX3 proteins [7]. Accordingly, certain DEAD box proteins may have roles in maintaining HIV latency. If this reasoning is correct, then the selective targeting of such DEAD box factors might offer another means of ending HIV latency and for depleting latent HIV reservoirs. The DEAD box proteins and other RNA helicases may therefore represent important cellular factors that can be manipulated to alter viral replication in several therapeutically useful ways.

Cellular genes may be differentially expressed during viral replication for many different reasons. Differential expression of cellular genes may conceivably occur because of viral actions on the host cell designed to optimize the cell for viral replication, because of cellular responses to infection aimed at inhibiting viral infection, or may be fundamentally unrelated to key aspects of viral replication. However, the findings that several DEAD box protein genes are differentially expressed during HIV replication, together with the recently published observations that two DEAD box genes, DDX3 and DDX1, exhibit differential expression during HIV replication and have important functions in HIV replication lend additional credence to the hypothesis that a careful, large scale study of differentially expressed cellular genes can provide insights into host cell factors involved in viral replication and pathogenesis. Future studies may reveal additional human co-factors for HIV replication. These cellular co-factors many represent important new therapeutic targets.

## Competing Interests

The authors declare that there are no competing interests.

## Authors' Contributions

VK designed and performed the experimental work and the data analysis. SZ directed and coordinated the study and participated in the data analysis. VK and SZ wrote the manuscript.
